# Supplementary material for: Population immunity to clade 2.3.4.4b H5N1 is dominated by anti-neuraminidase antibodies
Source: mBio. 2026 Apr 13;17(5):e00445-26. doi: 10.1128/mbio.00445-26 (PMC13170320; doi:10.1128/mbio.00445-26)
Supplement: Supplemental Material — Figures S1 and S2; Tables S1-S3. [file mbio.00445-26-s0001.docx]

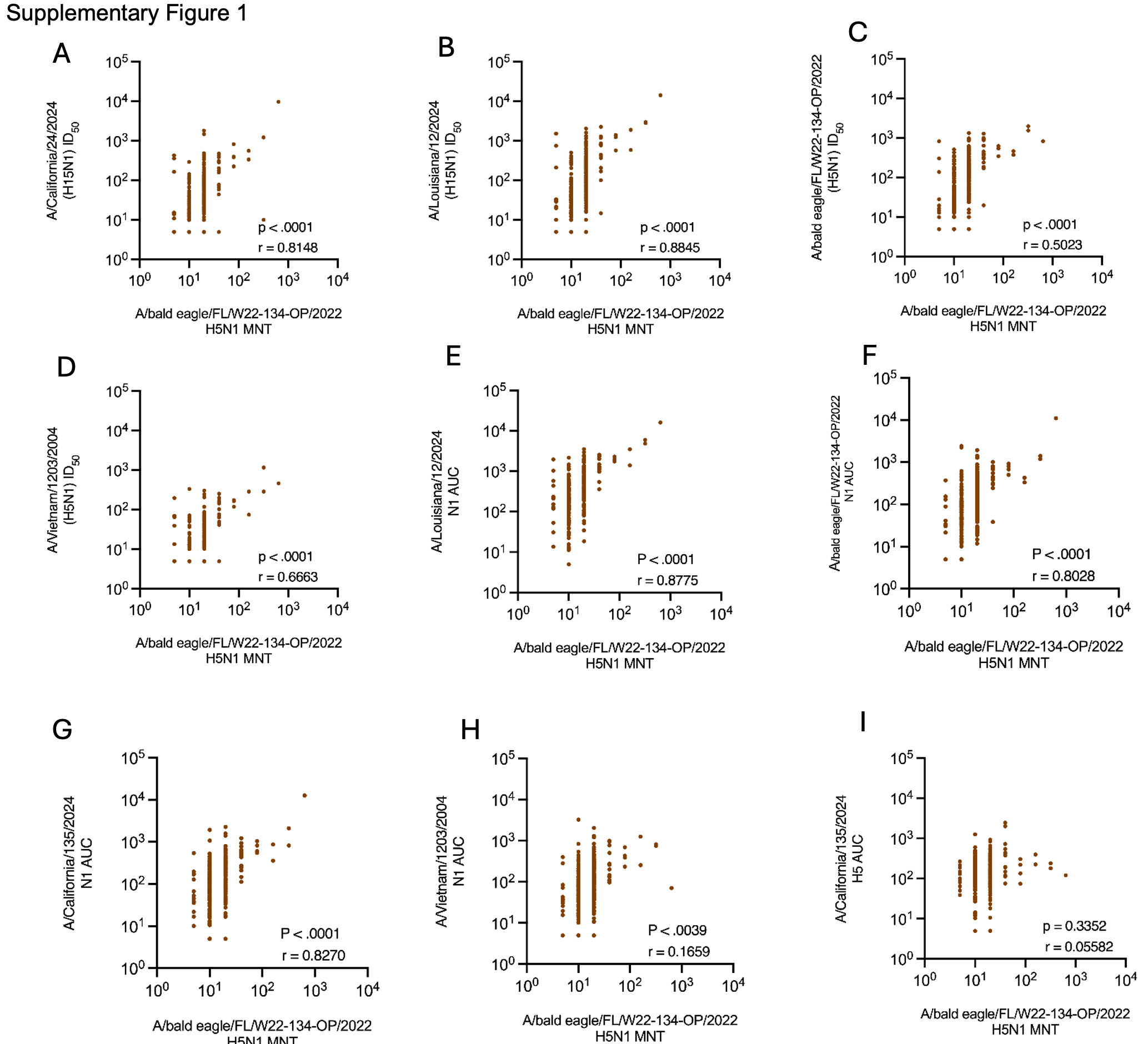
**Supplementary Fig. 1 Spearmen correlation of H5N1 microneutralization and antigen specific binding and NI antibodies**

(**A-D**) Spearmen correlation analysis was performed H5N1 microneutralization titers and H5N1 viruses NI antibodies (**E-H**) Spearmen correlation analysis was performed H5N1 microneutralization titers and N1 NA binding antibodies of H5N1 viruses. (I) Spearmen correlation analysis were performed H5N1 microneutralization titers and HA binding antibodies of H5N1 viruses. Correlation coefficient (r) and significance value (p) are indicated above the x axis.


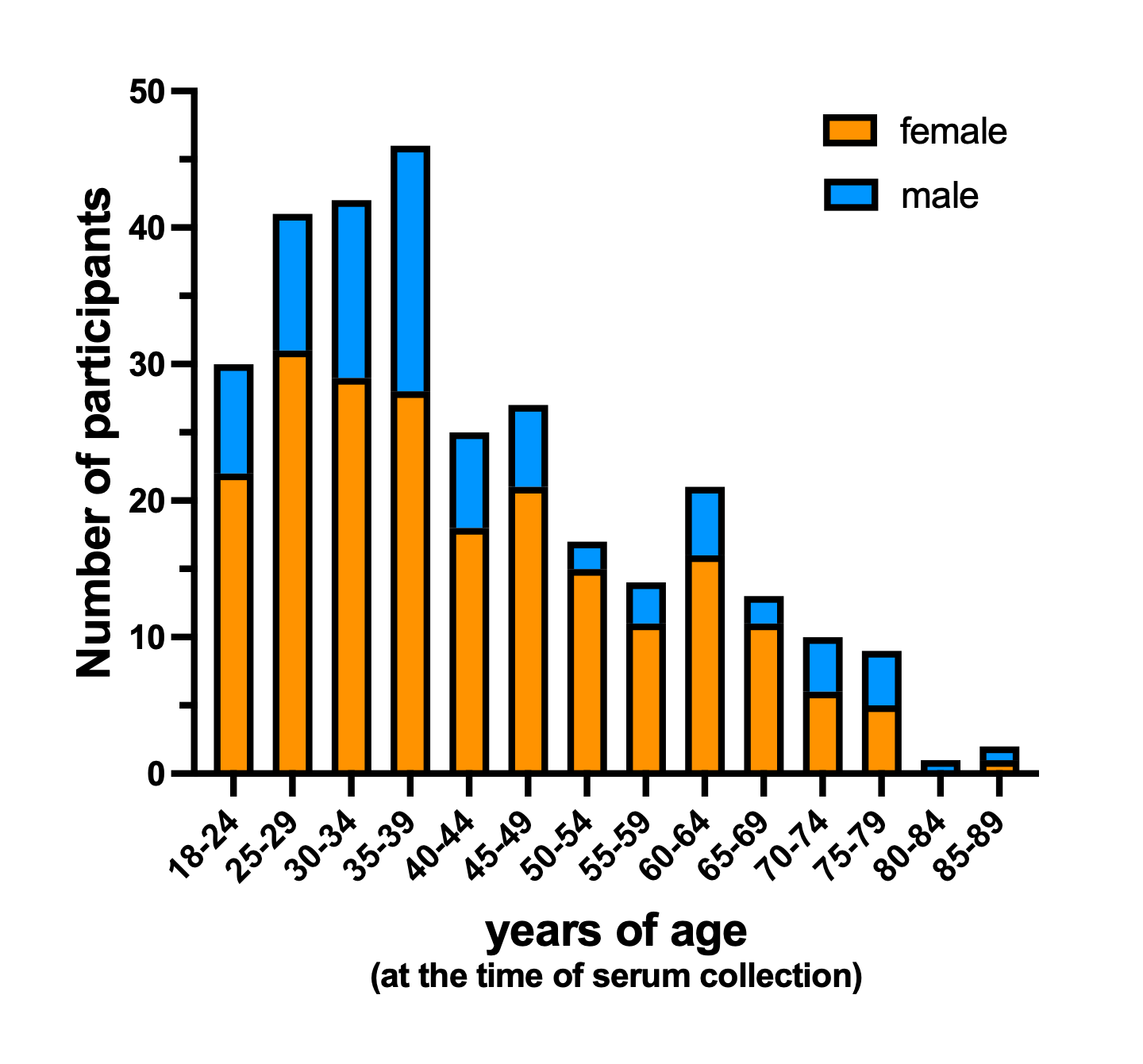


**Supplementary Fig. 2 Age and sex distribution of individuals from whom sera were analyzed.**

**Table S1 Detailed description of samples used (collected between February 2024 and April 2025)**

| **Participant Metadata (n=300)** | |
| --- | --- |
| **Age at Baseline** | |
| **Median [Range]** | 38 [18-85] |
| **Sex at Birth** | |
| **Female** | 214 (71%) |
| **Male** | 84 (28%) |
| **Not Reported** | 2 (1%) |
| **Gender** | |
| **Woman** | 211 (70%) |
| **Man** | 82 (27%) |
| **Non-Binary** | 2 (1%) |
| **Other** | 3 (1%) |
| **Not Reported** | 2 (1%) |
| **Race** | |
| **American Indian or Alaska Native** | 2 (1%) |
| **Asian** | 56 (19%) |
| **Black or African American** | 50 (17%) |
| **Middle Eastern or North African** | 1 (0%) |
| **White** | 16 (5%) |
| **More than One Race** | 130 (43%) |
| **Other** | 18 (6%) |
| **Not Reported** | 27 (9%) |
| **Ethnicity** | |
| **Hispanic or Latino** | 61 (20%) |
| **Not Hispanic or Latino** | 221 (74%) |
| **Not Reported** | 18 (6%) |
| **Flu Vaccine 2023-24 Season?** | |
| **Yes** | 128 (43%) |
| **No** | 103 (34%) |
| **Unknown** | 69 (23%) |
| **Flu Vaccine 2024-25 Season?** | |
| **Yes** | 148 (49%) |
| **No** | 80 (27%) |
| **Unknown** | 72 (24%) |
| **Flu Infection 2023-24 Season?** | |
| **Yes** | 6 (2%) |
| **No** | 225 (75%) |
| **Unknown** | 69 (23%) |
| **Flu Infection 2024-25 Season?** | |
| **Yes** | 3 (1%) |
| **No** | 219 (73%) |
| **Unknown** | 78 (26%) |

**Table S2: Percent amino acid identity of HA sequences of A(H5N1) and seasonal A(H1N1)**

|  | A/Cal/04/09 H1 | A/VN/1203/04 H5  clade 1 | A/Cal/135/24 H5 clade 2.3.4.4b | A/Guna/17SF003/16 H7 |
| --- | --- | --- | --- | --- |
| A/Cal/04/09 H1 | 100 | 64.0 | 63.7 | 40.9 |
| A/VN/1203/04 H5 clade 1 | 64.0 | 100 | 91.3 | 63.1 |
| A/Cal/135/24 H5 2.3.4.4b | 63.7 | 91.3 | 100 | 62.5 |
| A/Guna/17SF003/16 H7 | 40.9 | 63.1 | 62.5 | 100 |

|  | ****A/Cal/04/09 N1**** | ****A/VN/1203/04 N1****  ****clade 1**** | ****A/BE/FL/22 N1 clade 2.3.4.4b****  ****(B1.1)**** | ****A/Cal/135/24 N1 clade 2.3.4.4b (B3.13)**** | ****A/LA/12/24 N1 clade 2.3.4.4b (D1.1)**** | ****A/Gull/NS/23 N5****  ****(A6)**** | ****A/Wash/2148/25 N5****  ****(A6)**** | ****A/AH/1/13 N9**** |
| --- | --- | --- | --- | --- | --- | --- | --- | --- |
| ****A/Cal/04/09 N1**** | 100.0 | 72.3 | 78.3 | 78.3 | 83.3 | 59.1 | 59.1 | 46.0 |
| ****A/VM/1203/04 N1**** | 72.3 | 100.0 | 87.5 | 87.5 | 83.0 | 61.0 | 61.0 | 47.2 |
| ****A/BE/FL/22 N1**** | 78.3 | 87.5 | 100.0 | 99.0 | 88.3 | 63.0 | 63.0 | 47.2 |
| ****A/Cal/135/24 N1**** | 78.3 | 87.5 | 99.0 | 100.0 | 88.3 | 63.0 | 63.0 | 46.1 |
| ****A/LA/12/24 N1**** | 83.3 | 83.0 | 88.3 | 88.3 | 100.0 | 62.5 | 62.5 | 46.2 |
| ****A/Gull/NS/23 N5**** | 59.1 | 61.0 | 63.0 | 63.0 | 62.0 | 100.0 | 99.0 | 41.0 |
| ****A/Wash/2148/25 N5**** | 59.1 | 61.0 | 63.0 | 63.0 | 62.5 | 99.0 | 100 | 41.0 |
| ****A/AH/1/13 N9**** | 46.0 | 47.2 | 47.2 | 46.1 | 46.2 | 41.0 | 41.0 | 100 |

**Table S3: Percent amino acid identity of NA sequences of A(H5N1) and seasonal A(H1N1)**
